# Bees with attitude: the effects of directed gusts on flight trajectories

**DOI:** 10.1242/bio.034074

**Published:** 2018-08-22

**Authors:** Timothy Jakobi, Dmitry Kolomenskiy, Teruaki Ikeda, Simon Watkins, Alex Fisher, Hao Liu, Sridhar Ravi

**Affiliations:** 1 School of Aerospace Mechanical and Manufacturing Engineering, RMIT University, Melbourne, 3083, Australia; 2 Japan Agency for Marine-Earth Science Technology (JAMSTEC), Yokohama-shi, 236-0001, Japan; 3 Graduate School of Engineering, Chiba University, Chiba-shi, 263-8522, Japan

**Keywords:** Flapping flight, Gusts, Insect body dynamics, Flight control

## Abstract

Flight is a complicated task at the centimetre scale particularly due to unsteady air fluctuations which are ubiquitous in outdoor flight environments. Flying organisms deal with these difficulties using active and passive control mechanisms to steer their body motion. Body attitudes of flapping organisms are linked with their resultant flight trajectories and performance, yet little is understood about how isolated unsteady aerodynamic phenomena affect the interlaced dynamics of such systems. In this study, we examined freely flying bumblebees subject to a single isolated gust to emulate aerodynamic disturbances encountered in nature. Bumblebees are expert commanders of the aerial domain as they persistently forage within complex terrain elements. By tracking the three-dimensional dynamics of bees flying through gusts, we determined the sequences of motion that permit flight in three disturbance conditions: sideward, upward and downward gusts. Bees executed a series of passive impulsive maneuvers followed by active recovery maneuvers. Impulsive motion was unique in each gust direction, maintaining control by passive manipulation of the body. Bees pitched up and slowed down at the beginning of recovery in every disturbance, followed by corrective maneuvers which brought body attitudes back to their original state. Bees were displaced the most by the sideward gust, displaying large lateral translations and roll deviations. Upward gusts were easier for bees to fly through, causing only minor flight changes and minimal recovery times. Downward gusts severely impaired the control response of bees, inflicting strong adverse forces which sharply upset trajectories. Bees used a variety of control strategies when flying in each disturbance, offering new insights into insect-scale flapping flight and bio-inspired robotic systems.

This article has an associated First Person interview with the first author of the paper.

## Abstract (precis)

**Summary:** The effect of atmospheric gusts on the flight trajectories of bumblebees, reporting motion of flight influenced by gusts along with flapping-enabled control strategies that could be necessary elements of flight at this scale.

## INTRODUCTION

Insects display a remarkable ability to engage in nimble control over their trajectory and attitude during flight. These flight characteristics have inspired great scientific endeavors into flapping wings, developing our knowledge of valuable concepts for flight such as unsteady lift mechanisms (Ellington et al., 1996; Dickinson et al., 1999; Sane, 2003), control capabilities (Sane and Dickinson, 2001; Deng et al., 2006), stability (Cheng and Deng, 2011; Ristroph et al., 2013) and underlying wing functions (Wootton, 1992; Usherwood and Ellington., 2002; Zhao et al., 2010). Studies on specific features of flying organisms have provided intelligent inspiration in the field of robotic design (Nakata et al., 2011; Ma et al., 2013; for review, see Shyy et al., 2016). However, most studies treat the flow environment as smooth, a major divergence from its true unsteady form in almost all flying scenarios.

The unpredictable conditions of the lower level of the atmosphere are ever-present. For those flying systems that are smaller in scale, the complex arena contains airflow that is highly changeable in strength and structure. Even away from local effects such as wakes of structures and vegetation, the wind is highly turbulent (Watkins et al., 2010). All flying animals use flapping wings rather than fixed wings to produce the aerodynamic forces necessary for flight. A number of studies have found flapping to be more effective in overcoming the effects of atmospheric conditions. As opposed to fixed and rotary wings, flapping wings make use of highly unsteady flow structures such as dynamic stall and wake capture. This allows flapping wings to operate at low velocities which permit precise control maneuvers in hover. Flapping at Reynolds numbers relevant to this scale has shown positive effects in turbulence aided by LEV formation in elevated turbulence (A. Fisher, PhD thesis, RMIT University, 2013), while flapping has also been shown to overcome the effects of vortices (Ortega-Jimenez et al., 2013; Ravi et al., 2015) and gusts due to the formation of unsteady aerodynamic mechanisms (Fisher et al., 2016; Viswanath and Tafti, 2010). Literature regarding the effects of these conditions on the dynamics of flapping flyers and the essential flight control behaviors which may assist in dealing with them is scarce.

Gusts and other atmospheric fluid structures are frequently referred to in the literature as damaging conditions which impede the control performance of small aerial systems (Watkins et al., 2006; Ravi et al., 2015). While turbulence and structured flow vortex streets caused by object wakes are significant on the broad scale, isolated gusts could be a critical element of the unsteady local flight aerodynamic condition at smaller scales and thus are particularly relevant to insects. In cluttered environments, studies refer to the existence of vortical wakes, particularly von Karman streets, that present insects with severe control challenges (Ravi et al., 2013; Ortega-Jimenez et al., 2013). The approximate scale of these vortices relevant to insects can be on the order of a few centimeters, matching the comparable scale of many insect wings. Relative to a flying insect navigating through one of these wakes, the adverse flow that interacts with the wings could be adequately described by a discrete gust containing local flow that meets the airborne surfaces predominantly in a singular direction.

A foraging mission for an insect on a typical day will involve sudden transitions from regions of varying air states in the low altitude region of the atmospheric boundary layer (ABL) (Watkins et al., 2010), flying across treacherous winds to a source of food or pollen, narrowing in on small and often dynamic landing sites (Chang et al., 2016), gleaning nectar on an often unstable platform and then navigating back for the return trip. The most aerodynamically challenging of these events is likely to be step changes between air scenarios and pinpointing a landing while complex flight maneuvers are impeded by unpredictable airflows. Approaching any solid object will involve traversing across shear layers and wind near the surface (Krujit et al., 2000), where the depth of the shear layer will likely be close to a few characteristic insect-wing dimensions (Crall et al., 2016). Local wakes including vortex shedding from surrounding vegetation could impinge on the insect from any orientation. The flow field is thus dominated by small changes from the wakes and vortices shed from plant structures (Stull, 1988).

The magnitude of the atmospheric wind varies with elevation, terrain and climatic conditions. It can vary from zero on calm days (typically 5% of the time for non-cyclonic areas) to extreme, typified by the one-hundred-year return wind speed. It has been shown that in the few meters from the Earth's surface the most likely speed is 3 m s^−1^ and that for 95% of the time the speed is less than 10 m s^−1^ (for details see Watkins et al., 1995). In this study, a single gust speed of 5 m s^−1^ serves as a relevant basis for examining flying insects.

Recent studies demonstrate that organized body orientation maneuvers interlace the translatory motions observed among many flying insects in turning flight: where roll axis rotations manipulate forces for changing bearing (Ristroph et al., 2012; Wang et al., 2003; Zeyghami and Dong, 2015 preprint); in flight initiation where body attitude adjustments could aid wing angles for favorable aerodynamic performance (Bode-Oke et al., 2017), and landing in which voluntary body manipulation relative to the nearby surface assists control (Evangelista et al., 2010); and in hovering for stability purposes (Sun, 2014) as well as in forward constant-speed flight to carry out operations such as casting (Ravi et al., 2013). Attitude manipulation for control has also been found in involuntary (disturbance negotiation) flight scenarios in turbulence (Combes and Dudley, 2009), gusts (Vance et al., 2013) and vortices (Ravi et al., 2013; Ortega-Jimenez et al., 2013) and inertial perturbations (Ristroph et al., 2010) where insects passively generate restoring forces that influence body attitude. The understanding of the effects and interactions that gusts exhibit on the body motions of insect-scale flapping flyers has not been studied extensively. Attitude adjustments that can boost control of translatory motions in common flying environments could reveal important information regarding control mechanisms for all insect-scale flight.

We explored the flight dynamics of flying insects in strong gusts from three orthogonal directions. The sequences of trajectory and attitude changes in six degrees of freedom were tracked to measure the influence of the gusts on the flight trajectories. We recorded bumblebee flight paths and extracted velocities and accelerations from these data to derive dynamic information. Statistically significant comparisons found by via paired *t*-tests were gathered from the data to build on our understanding of the effect that gusts have on centimeter-scale flapping flight.

## RESULTS

### Flight phases and attitude maneuvers

Bees negotiated gusts uniquely when flying through each of the three gust directions. All bees displayed a combination of impulsive attitude maneuvers – those caused involuntarily by the sheer force of the gust – followed by a series of recovery attitude maneuvers – those performed voluntarily in resistance to the gust in pursuit of recovery. To determine the time length in each of these two phases, the moment of gust entry was taken as the start of the impulsive phase. We then computed an attitude acceleration curve to know the exact time bees initiated the recovery phase (see Fig. S1 for details). We termed these two distinct chapters of flight the ‘impulsive phase’ and the ‘recovery phase’, respectively. Attitude maneuvers were unique in each gust direction, yielding interesting roll, pitch and yaw signatures that highlight potential strategies for governing control.

Bees flew in the center of the tunnel at 0.5 m s^−1^ (µ=0.50±0.03 m s^−1^) having a mean neutral pitch inclination angle of 26° (µ=25.7±2.41°) on approach to each gust. The first sign of a bee entering a gust was usually visible by a sharp deflection in the antennae.

Attitude maneuvers in the impulsive phase of all gust directions were always in the direction of ‘push’ caused by the gust. Recovery maneuvers usually opposed the force of the gust and involved corrective efforts to regain stability towards the original trajectory. Two impulsive maneuvers were detected as bees flew through the sideward gust – one in roll and one in yaw. Bees were forced to roll away from the gust (port side down) by an average of 29° (µ=−28.6±10.9°) while first experiencing sideward gusts (Fig. 2A). A clockwise yaw maneuver of mean 20° (µ=−20.4±5.9°) (turning away from the gust about Z, the vertical axis) occurred concurrently. Body acceleration in the direction of the gust only occurred after the first impulsive roll maneuver.

Bees flying into upward gusts exhibited smaller kinematic disruption than those flying into sideward gusts. Bees pitched up approximately 33° above the neutral pitch angle as they flew through the upward flow (Fig. 3B). The time of maximum pitch up in upward gusts occurred at varied intervals after the time of gust entry, and hence the mean pitch curve shown in Fig. 2B for the upward gusts is more diluted than that of the sideward (Fig. 2A) and downward gusts (Fig. 2C). This pitch up maneuver is shown to be significant compared to deviations observed in steady flight (Table S2). Rotations in roll and yaw followed with no distinct pattern leading into recovery. In the recovery phase, bees pitched back down beyond the original neutral position. This was probably to direct the mean lift vector forward and produce forces for increasing forward flight speed by way of the ‘helicopter’ control model. The pitch down maneuver occurred alongside corrective rotations in roll and yaw that oscillated between ±20° throughout the rest of recovery.

The response of bees flying through downward gusts was more erratic than that of the upward gust. A sharp pitch down maneuver of approximately 14° (µ=14.3±6.80°) below neutral was observed during the impulsive phase when bees began to intercept the downward flow of air (Fig. 2C). During the gust-induced fall, bees extended their legs and produced a sharp pitch up maneuver of about 47° (µ=47.1±4.10°) to mark the beginning of the recovery phase. Bees took a pitch down maneuver back to neutral during the recovery phase which was interlaced with large roll (±40°) and yaw (±11°) corrections. By the end of recovery, bees had regained steady attitudes but trajectories usually remained displaced.

Attitude deviation was generally larger around the roll axis in all gust directions as shown by the comparison of attitude deviation means represented in (Fig. 3B). The horizontal gust (sideward) directly affected bees about the roll and yaw axes whereas the vertical gusts (upward, downward) affected bees about the pitch axis. These axes of direct gust-influence arise due to the respective incidence angles of the gust on the bee and resultant orthogonal aerodynamic forces across gust directions. A large difference arose between the pitch maneuvers in the two vertical gusts. The dissimilarity between these two cases should only be the gust force direction, yet we found that mean impulsive pitch maneuvers were 10° greater in the downward gust (pitch down) relative to the upward gust (pitch up) (*P*=0.021) (Fig. 2). In sideward gusts, mean impulsive pitch maneuvers deviated similarly, actually exceeding the upward gust by a few degrees. While the sequence of attitudes in the impulsive phase was governed by the gust direction (i.e. all maneuvers in the push direction of the gust), we found that bees showed a pattern for regaining control in the recovery phase of flight through all gust directions. All bees pitched up in response to the disturbance at the start of recovery regardless of gust direction; this was followed by oscillatory corrective adjustments to roll and yaw whose magnitude varied between gusts and appeared to assist in the body motions.

### Trajectory and velocity

**Fig. 1.**
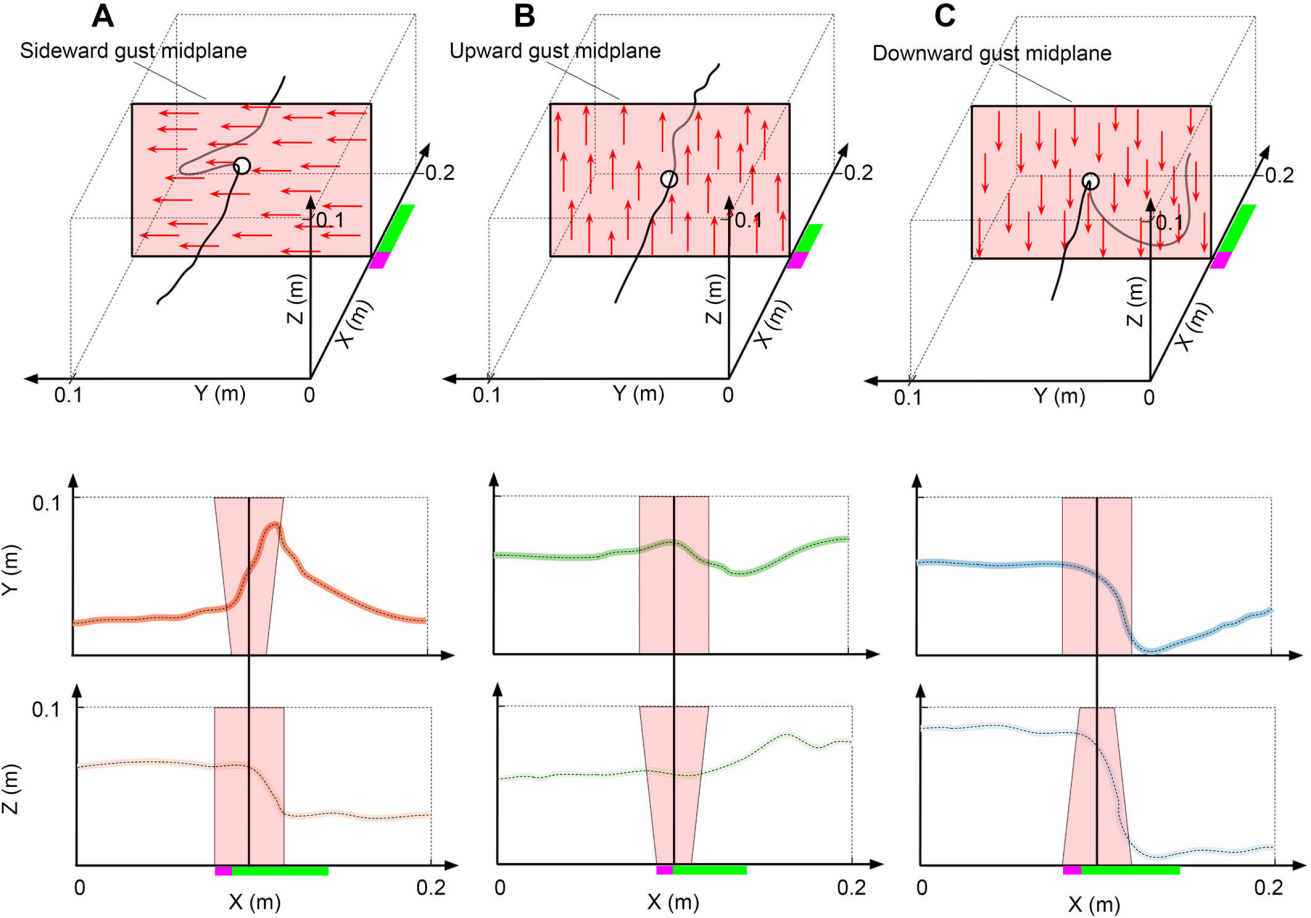
**Bumblebee flight trajectories.** Example flight trajectory through the calibrated volume in: (A) a sideward gust, (B) an upward gust and (C) a downward gust. Side and top views of trajectory are displayed below in terms of Z and Y respectively. The planes shaded in red in the first row of panels (A,B,C) are perpendicular to the plane represented in Fig. 9 such that they resemble the middle plane of the gust.

Sideward gusts perturbed the maximum lateral position of bees by 48 mm (µ=48.3±3.2 mm), while bees in upward and downward gusts were displaced vertically by 32 mm (32.1±2.3 mm) and 53 mm (µ=52.9±5.7 mm) respectively (Fig. 3) (Table S1). Individual maneuvers during the impulsive phase were followed by matching trajectories. For example, the impulsive portside roll/yaw maneuver was immediately followed by leftward motion in the lateral direction and the impulsive upward/downward pitch maneuvers (in vertical gusts) were followed by motion in the vertical axis. The downward gust forced bees into a very rapid nosedive which caused a loss of altitude that exceeded the translational deviations of both upward and sideward gusts (Z_down_*–*Z_up_, *P*=0.002; Z_down_*–*Z_side_, *P*<0.001) (Fig. 3A) (Table S2). This altitude change was around three times greater than the altitude change in its rival vertical gust (upward). In the axes orthogonal to sideward gusts, bees generally lost altitude (µ=5.36±7.61 mm) during the sideward disturbance. The maximum horizontal deviations in the vertical gusts were 31 mm (µ=30.9 mm±2.89 mm) in the upward gust and 28 mm (µ=28.1 mm±2.27 mm) in the downward gust – comparable to the magnitude of vertical deviation in the same gusts (Fig. 3) (Table S1). After the perturbation, bees returned to the x-y center of the tunnel in all gust directions, but bees didn't seek to return to the x-z center in the downward gust (Fig. 1).

**Fig. 2.**
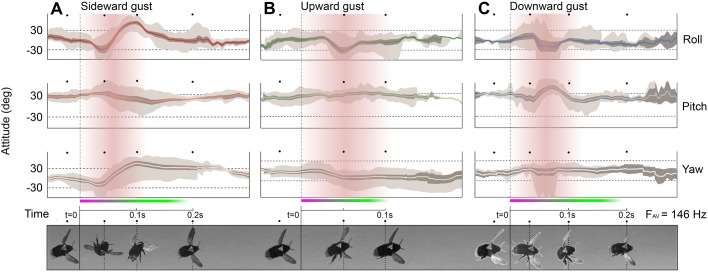
**Bumblebee flight attitudes.** Mean attitude maneuvers of bees flying through (A) sideward gusts, (B) upward gusts and (C) downward gusts. Data are plotted against absolute time referenced to the time instance at which bees first enter the gust on the time axis. Gusts are illustrated in red where fading color represents the time at which bees were less likely to be within the gust. Dark shading represents the standard error of the mean attitude. Light shading shows the maximum and minimum angular displacements recorded. Response phases are shown in a purple (impulsive) zone and a green (recovery) zone. Corresponding image snapshots of bees during major maneuvers are represented sequentially below each set of plots (D).

**Fig. 3.**
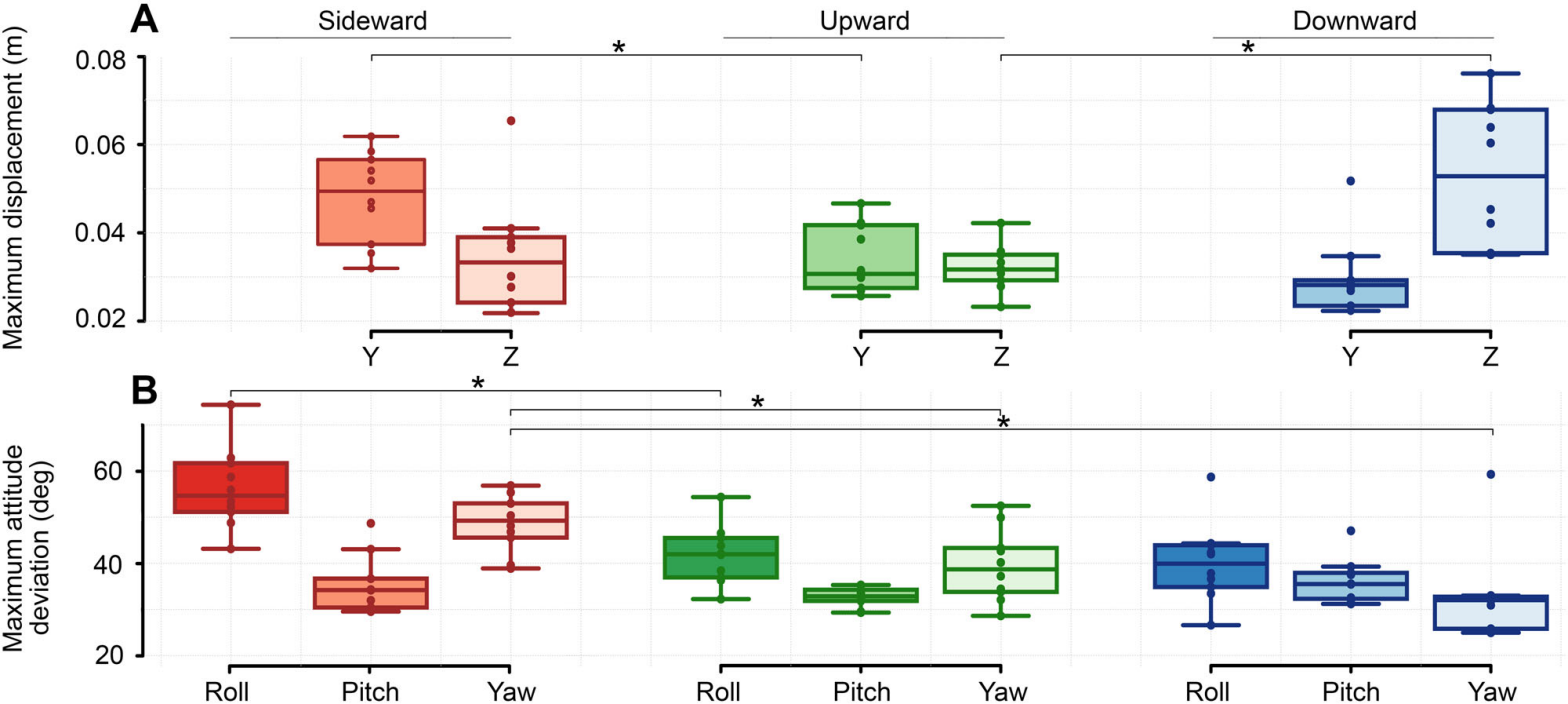
**Flight displacements.** Box plots of (A) maximum translational displacements and (B) maximum rotation deviations for bees flying in three different gust directions. Box plots are compared using paired *t*-tests (*n*=10) in each set and shown by asterisks where the *P*-value between two independent samples is less than 0.05 (see Table S1 for complete list data).

**Fig. 4.**
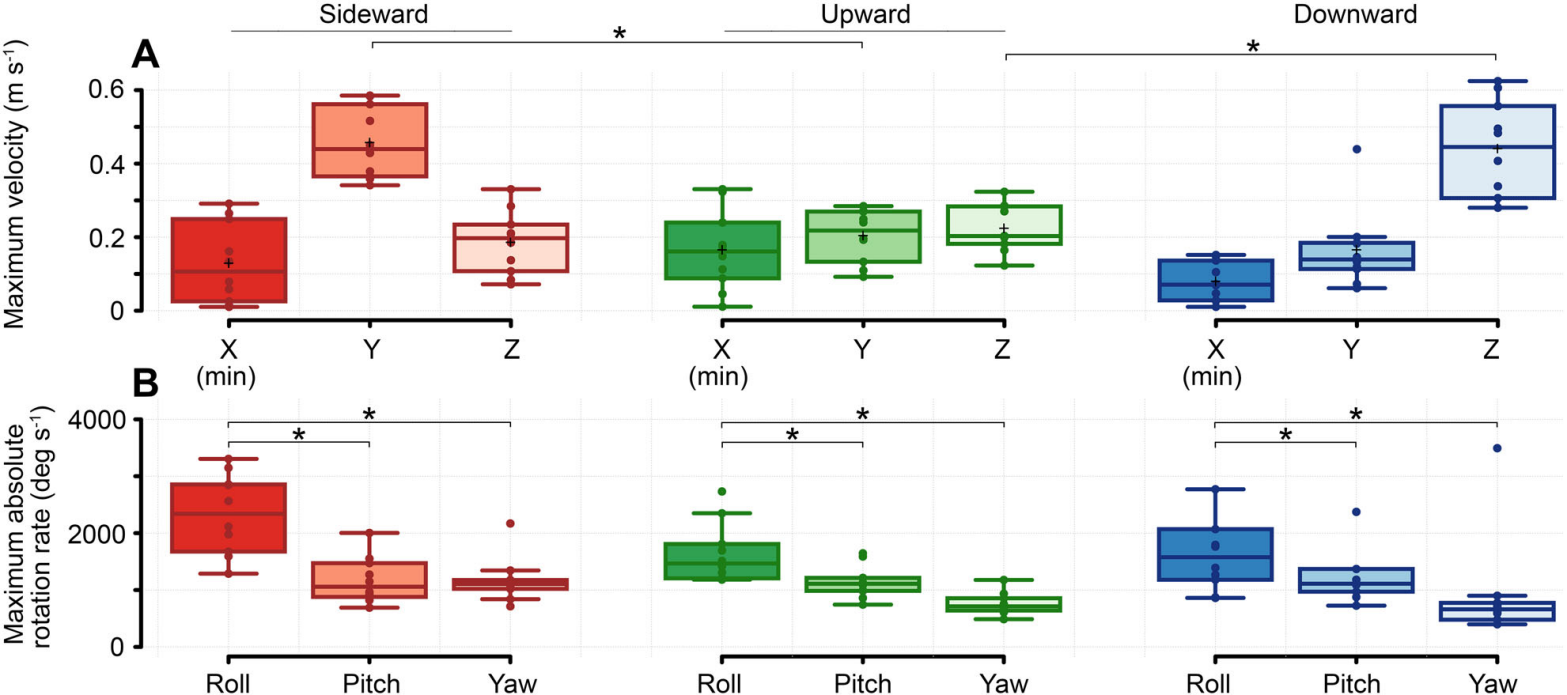
**Flight velocities.** Box plots of (A) maximum translation rates and (B) maximum rotation rates in three gust directions. Note that box plots for X velocity show minimum values. All other plots are maximum values which were taken after applying filters to reduce error arising from the numerically differentiated data (see Fig. S1). Significance is determined by using paired *t*-tests (*n*=10) for all sets.

In all three gust directions, attitude changes occurred most rapidly around the roll axis for all flights (Fig. 4). Sideward gusts caused bees to roll at a maximum rate of 2300° s^−1^ (µ=2311±254° s^−1^) and yaw at 1100° s^−1^ (µ=1092±82° s^−1^) (Fig. 4B) (Table S1). On average, upward gusts caused pitch to change at a maximum rate of 1000° s^−1^ (µ=1045±89° s^−1^) and downward gusts caused an angular rate at an average value of 1100° s^−1^ (µ=1058±93° s^−1^), nearly half of the gust-induced roll rates recorded in the sideward gust (Ṙ_side_*–*Ṗ_up_, *P*=0.001; Ṙ_side_*–*Ṗ_down_, *P*=0.002) (Table S2). Maximum roll rates in the upward and downward disturbances occurred at mean magnitudes of 1500° s^−1^ (µ=1514±133° s^−1^) and 1700° s^−1^ (µ=1684±209° s^−1^) respectively (Fig. 4) (Table S1).

All bees slowed down from their mean forward velocity of 0.50 m s^−1^ after encountering a gust. Bees in sideward gusts slowed to a minimum of approximately 0.13 m s^−1^ (µ=0.13±0.04 m s^−1^) while downward gusts slowed to a speed of around 0.07 m s^−1^ (µ=0.07±0.02 m s^−1^) (Fig. 4A). This value is significantly less than that of the upward gust, which slowed to a mean velocity of 0.17 m s^−1^ (µ=0.17±0.03 m s^−1^) (Table S1). Bees seemed to slow down according to the apparent gust severity that we observed for each gust. After passing through gusts, bees gradually regained forward velocity up to the original mean velocity. Lateral velocities in the global coordinate system peaked at about 0.5 m s^−1^ (µ=0.43±0.03 m s^−1^) in sideward gusts, greatly exceeding the longitudinal velocities in the disturbance. The max upward velocity in upward gusts was comparable to the lateral velocities which averaged at around 0.2 m s^−1^ (µ=0.21±0.03 m s^−1^), marking only subtle changes in velocity between the three axes. The downward gust produced the greatest gust-induced velocity, seeing a global downward velocity of approximately 0.5 m s^−1^ (µ=0.44±0.04 m s^−1^) – comparable to the lateral velocities caused in the sideward gust (Ż_down_*–*Ẏ_side_, *P*=0.93) but far greater than the upward velocities triggered by the upward gust (Ż_down_*–*Ż_up_, *P*<0.001) (Fig. 4A) (Table S2).

After arresting the effects of a gust, bees resumed travel through the tunnel. Bees spent a mean time of 0.18 s (µ=0.18±0.04 s) negotiating the sideward gust (Fig. 6A) from the moment of entry to a location 20 mm beyond the exiting side of the gust sheet. This location was based on the knowledge that bees complete recovery within 20 mm beyond the point of gust exit. Time required to travel through gusts differed greatly for the upward gust, requiring approximately 0.12 s (µ=0.12±0.05 s) to fly through, but compared similarly with the downward gust, which on average took 0.18 s (µ=0.18±0.03 s). These total transit times form the sum of impulsive and recovery times taken in the phases of each flight. The impulsive phases of flights through all gust directions were approximately equal in all cases at about 0.06 s (*t*_*i*__side_*–t*_*i*__up_, *P*=0.92; *t*_*i*__side_*–t*_*i*__down_, *P*=0.53; *t*_*i*__up_*–t*_*i*__down_, *P*=0.67) (Fig. 6B) (Table S2). Bees took the longest time to recover in the downward gust (µ=0.125±0.03 s) in a similar interval to sideward gusts (µ=0.121±0.04 s) (Table S1). The discrepancy with its upward rival (µ=0.051±0.05 s; *t*_*r*__up_*–t*_*r*__down_, *P*=0.001) exceeded a factor of two, showing that the noted lesser displacements in upward gusts take less time to fly through (Fig. 6B).

**Fig. 5.**
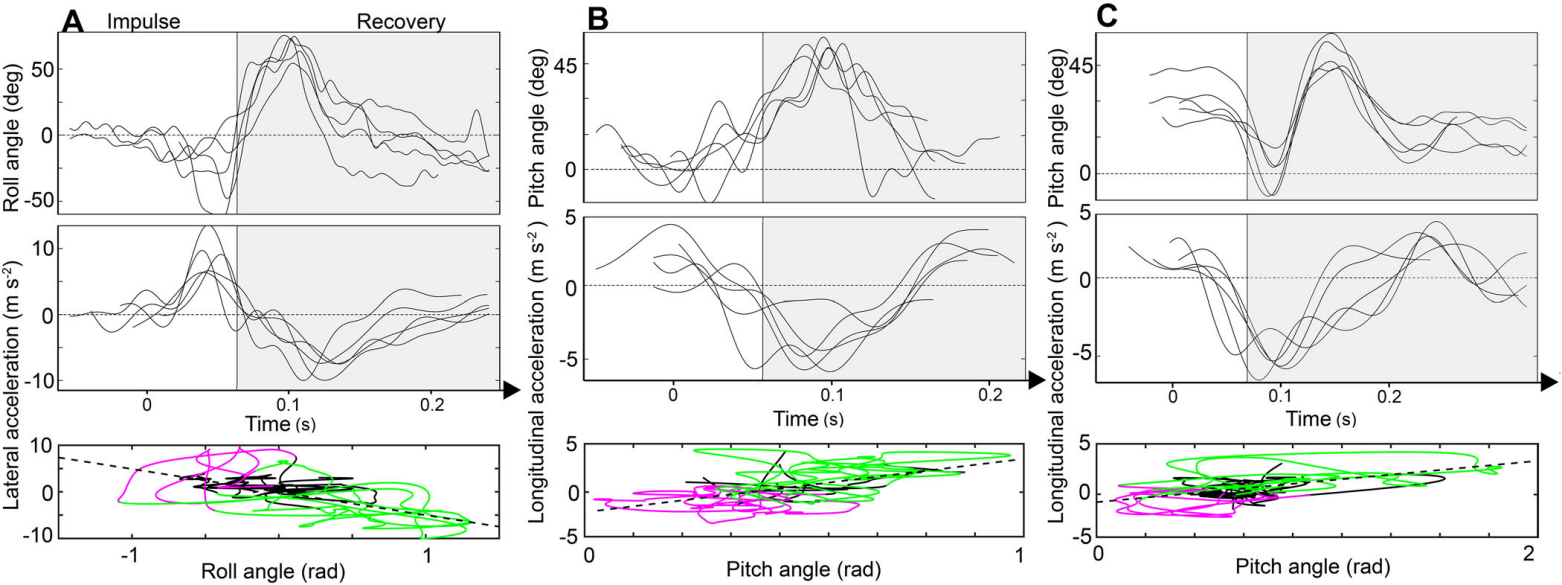
**Flight accelerations and attitudes.** Respective bee attitudes and accelerations of five individual bees in the horizontal plane. These are plotted against absolute time distinguished by the impulsive phase (white background) and recovery phase (grey background) in (A) sideward gusts, (B) upward gusts and (C) downward gusts. Regression lines are plotted for bee acceleration against corresponding attitude angle on the lower set of axes underneath, where flight phases are highlighted in purple (impulsive phase) and green (recovery phase). Continuous black lines represent the period of flight before entering the gust.

Regression lines for attitude deviations against corresponding accelerations in the horizontal plane were used to determine how body rotations precede control motions (Fig. 5). The impulsive roll maneuver in sideward gusts strongly correlated in a negative manner with lateral acceleration by a value of 0.50 (Fig. 5A) purely by the passive ‘sailboat’ model. Recovery roll attitude maneuvers correlated moderately with lateral acceleration by a value of 0.44 (compared to 0.32 in vertical gusts). Likewise in vertical gusts, we found a strong positive correlation between impulsive pitch maneuvers and longitudinal acceleration by 0.55 in upward gusts (Fig. 5B). In downward gusts, the more violent impulsive pitch maneuvers correlated strongly with longitudinal acceleration (0.61) meaning that bees had a greater disruption to oppose in recovery due to the larger disturbance produced by the downward gust (Fig. 5C). Recovery in all gusts was characterized by the pitch up maneuver along with longitudinal deceleration at an average positive correlation value of 0.52, showing that bees consistently used pitch attitude changes to manage the slowdown in response to the disturbances. The slope of the regression line in all cases could be expected to be about the magnitude of gravity (9.8 m s^−1^^2/rad) if altitudes were held constant. However, since altitudes were not stable, we calculated a significantly smaller regression line slope in the downward gust (4.9) compared to the upward gust (6.4) based on multiple regression analysis (*P*<0.05).

## DISCUSSION

**Fig. 6.**
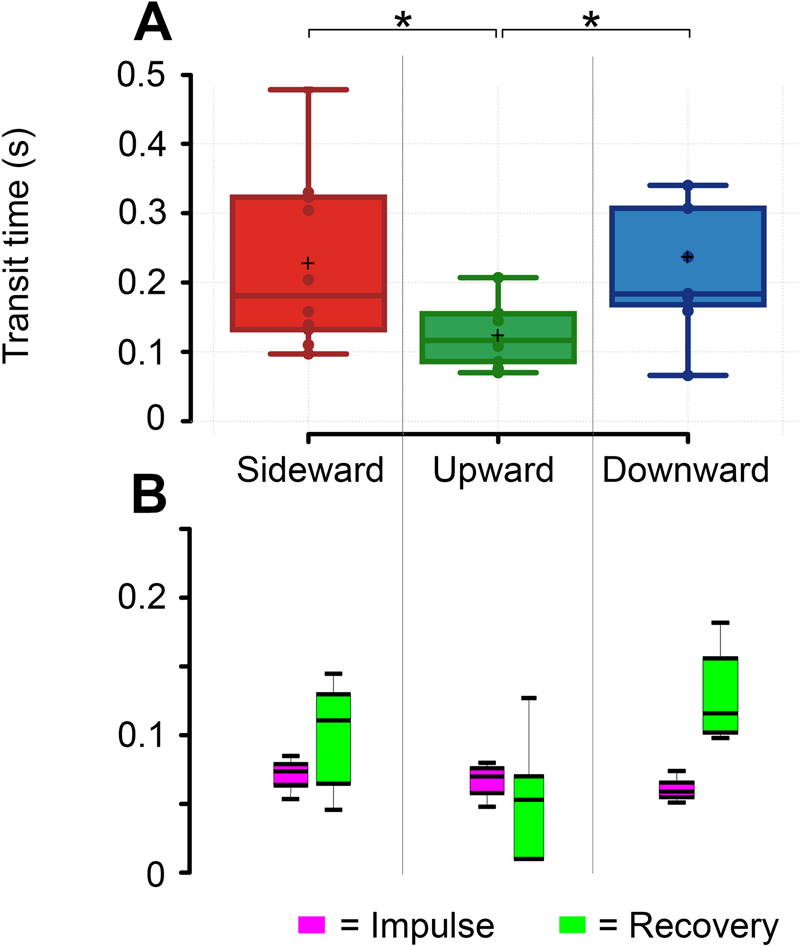
**Flight velocities** (A) Box plots showing time taken to transit through the entire disturbance caused by the gust. (B) Box plots of time taken through the individual impulse (purple) and recovery (green) phases in each of the gust directions. Asterisks are shown where the results of paired *t*-tests (*n*=10) indicate significance (*P*<0.05).

The results reveal that bees tackle gusts from different directions with varying levels of difficulty. Sideward and downward gusts are significantly more difficult for bees to fly through than upward gusts, shown by smaller magnitudes of nearly all dynamic quantities in upward gusts. Major differences in the responses from each gust direction arose mainly within the recovery period of flight. The large horizontal deviations in the vertical gusts (comparable to the gust-direction deviations in upward gusts) and the minimal vertical deviations in the sideward gust shows that bees used the lateral plane for control adjustments more than the vertical plane. These adjustments were usually characterized by interlaced roll and pitch attitude maneuvers correlated with subsequent flight paths. Impulsive maneuvers were endured for a roughly equivalent amount of time across each of the gust directions (Fig. 6B). The small differences here are too large to pinpoint any directional sensory time sensitivity between gusts. The recovery phase captured the differences between different gusts and these reflect the challenges with flying through certain directions of flow as indicated by the contrasting recovery time intervals. Energy expenditure is to some degree proportional to time in flight. We can thus infer that the approximate energetic cost of flight in each condition is significantly dissimilar across different gust flow directions. This shows that downward gusts issue a more damaging and energy-sapping challenge, significantly more so than upward gusts and comparable to sideward gusts, although in sideward gusts bees appeared to have a more ordered strategy for dealing with the disturbance. The independent response measures taken in each gust, both passively and actively, yield interesting strategies for control (Fig. 1).

In the upward and downward gusts, no differences in the disturbance exist between the two cases other than the direction of the gust relative to gravity. We speculate that previously unidentified aerodynamic interactions with flapping wings could occur when gusts strike the surface from above or from below. Flow phenomena have been shown to uniquely interact with flapping wing aerodynamics, confirming that certain conditions can be detrimental to flapping flight (Engels et al., 2016; Kolomenskiy et al., 2016 preprint; Ravi et al., 2016; Crall et al., 2016). Based on our data we postulate that gusts directed downward may interact with flow structures such as the LEV and wake effects at low Reynolds numbers. We see a distinct difference in the gradients of the regression lines (Fig. 6B,C). This shows that the lift force vector was more severely impaired in the downward gust, likely a result of the destabilizing effect of the gust on flow structures which are critical to maintain the flight forces necessary for insect flight. Some studies have previously hinted that downward gusts can interfere with the LEV of insects (Jones and Yamaleev, 2016, Jones and Yamaleev, 2012). In upward gusts, these mechanisms could be somewhat protected by the higher-pressure surface below. Our results indicate peculiar aerodynamic interactions between flying insects and gusts, for which further studies are required to uncover the underlying processes by which these occur. We aim to measure the reason for the discrepancies in future work with a representative robotic flapper that could help to solve these unknowns.

In the case of the sideward gust, bees always yawed towards the direction of the gust rather than continuing straight flight through the tunnel. It could be that bees are passively stable around the Z-axis as observed in hawkmoths (Nguyen et al., 2016). Recordings showed that bees consistently shifted mean wing stroke angles rearwards upon reaction of the disturbance. This rearward shift could cause a stabilizing moment about the Z-axis which assists the observed yaw motion (effectively converting the sideward gust into a frontal gust relative to the bee). Flapping with an average stroke angle behind the center of gravity of the body, bees can be expected to generate additional yaw torque as the wing collects forces from the gust at some distance behind the centre of gravity (CG). This behavior likens to the inherent passive stability of the tail of a conventional passenger aircraft or a feathercock. In addition, limb extensions manipulate stability by augmenting aerodynamic force acting on the body and thereby amplifying the resultant stabilizing body torques. Limb extensions also shift the center of gravity downwards causing a more ‘bottom-heavy’ distribution of mass (Liu et al., 2012), which is a known attribute for stable control in flying insects.

The tendency of bees to move in correspondence with their attitude is a method for control that enables flapping flyers to produce flight forces for motion in the horizontal plane. The ‘helicopter mode’ has been witnessed amongst bumblebees (Ravi et al., 2016, 2013), hawkmoths (Greeter and Hedrick, 2016) and fruit flies (Muijres et al., 2015) along with a number of bird species during routine flight maneuvers (Thomas and Taylor, 2001; Ros et al., 2011). This method of control and an idealized ‘sailboat’ model has been seen in bees struck by lateral flows (Ravi et al., 2016). Here, we show that the helicopter mode is employed throughout the sequence of maneuvers demanded by gust perturbations when the flow axis is directed sideward, upwards and downwards. The change in total force, which is also a key element of the helicopter model, was excluded due to estimation difficulties in unsteady flow. We consider this to be an acceptable simplification, and therefore the results may contain some small degree of inaccuracy that doesn't interfere with the main findings. In consideration of aerodynamic force magnitude modulations, in this study it is clear that bees used the helicopter mode of control during impulsive and recovery maneuvers when struck by gusts from all tested directions. When flying through steady air, bees used the helicopter mode to undergo side-to-side casting motions. This was also true in the impulsive phase of flight although bees did not have active control of their body motion due to limits to their reaction time. Rather, all resultant forces acting on the body were passively commanded by the interaction of the buffeting gust on the insect body, yet were nonetheless in agreement with the helicopter control model.

In steady flight, bees varied longitudinal forward-flight velocity along the tunnel between 0.22 m s^−1^ and 0.57 m s^−1^ (µ=0.5±0.03 m s^−1^). We found that gust entry velocity correlated with transit time by r=0.72. This demonstrates that the velocity of bees when entering a gust is a responsible factor in the severity of the resultant disturbance. This is likely due to the association of velocity with disturbance impulse time and the resistance of inertial changes by gust forces. For both of these reasons, bees may benefit from barging through gusts rather than taking it slow in the case of a single discrete disturbance. However, a trade-off arises between sensory detection time and gust impulse time. Bees that travel faster would have to travel through more of the unsteady, potentially dangerous flow conditions before assessing the threat it poses. On the other hand, bees that travel slower would receive greater aerodynamic impulses from the gust. Alternatively, it may be possible that bees merely undergo the observed motion due to the intrinsic stability of the system. However, the strong correlation between pitch maneuvers and deceleration is evidence to support that the observed braking is a voluntary decision. In this study, most bees seemed to cautiously slow down in favor of sensory awareness. Those few that surged through the gust were able to deal with the recovery briskly but this may not be the case for imperfect natural conditions less discrete than that of this study.

### Concluding remarks

This work studies a skillful natural flyer to identify several control-related behaviors during its flight through gusts. The results show that bumblebees tackle gusts from different directions in different ways, but always pitch up and slow down upon meeting each disturbance. Bees are shown to be more affected by downward gusts than upward gusts. Sideward gusts cause a disturbance similar in magnitude to downward gusts, though bees appear to have a robust method for overcoming the more common lateral hindrance. Bees yawed into the sideward gust, be it passively or actively, which increased the frontal component of flow, thereby augmenting aerodynamic force production and control. These control strategies are useful in uncovering the clever flight conducts of volant insects, while providing potentially useful bio-inspired ideas towards the development of similar scale flying machines.

## MATERIALS AND METHODS

### Experiment setup

**Fig. 7.**
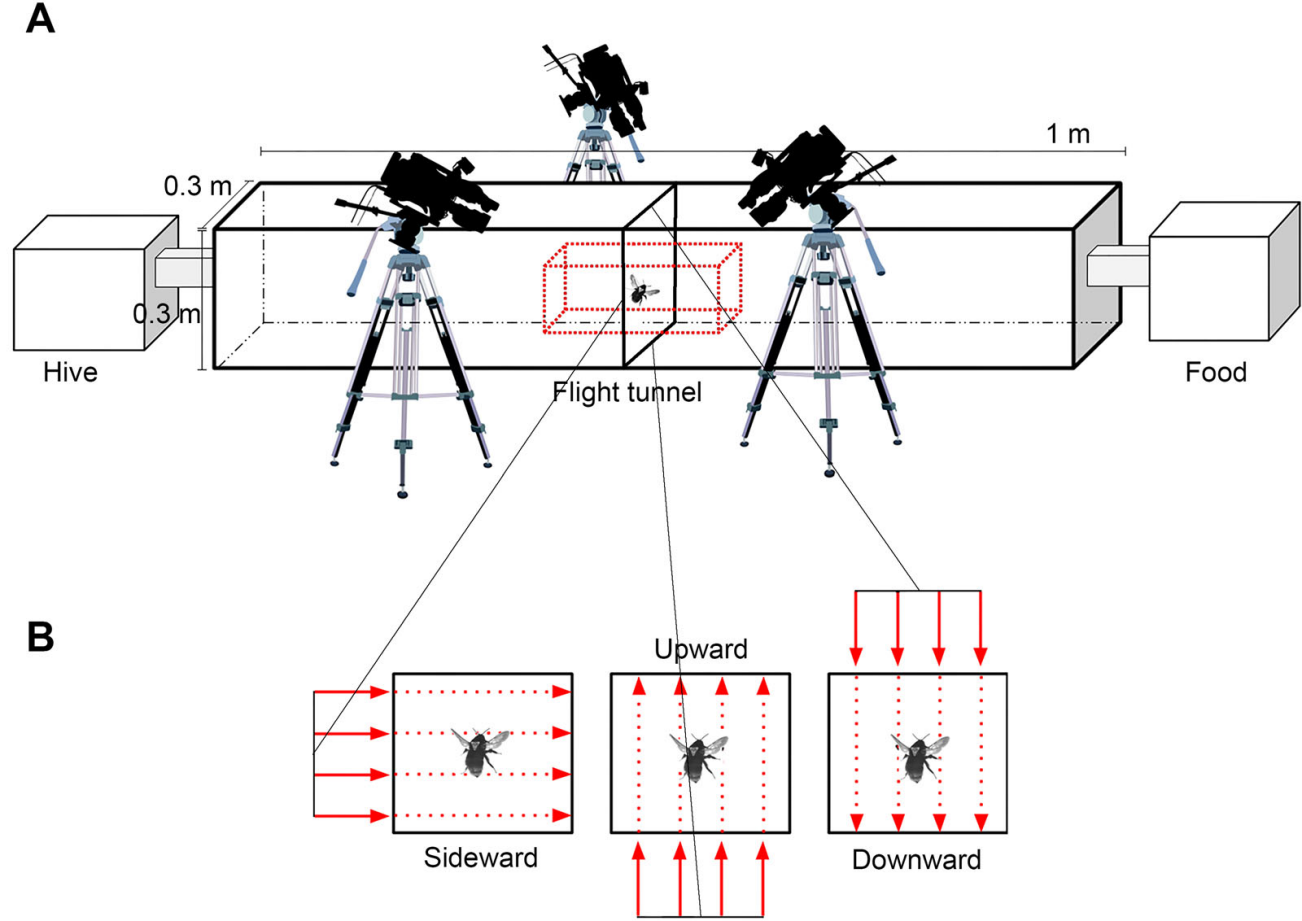
**Experiment layout.** (A) Setup of the bumblebee domain showing arrangement of the three interconnected sectors: the hive area, flight tunnel and feeding chamber. Configuration of recording equipment relative to the calibrated volume (represented by a red dotted prism around bee) shown in reference to the flight tunnel. (B) Cross-section views of the middle of the flight tunnel where identical gusts are directed sideward, upward and downward across this plane.

Bumblebees (*Bombus ignitus)* sourced from a commercial breeder (Koppert, distributed by Arysta LifeScience Asia, Tokyo, Japan, product name: Mini Polblack) were sustained in laboratory conditions. A foraging chamber of dimensions 1 m×1 m×0.8 m was accessible to the bees via a stagnant-air tunnel 1 m long, constructed from clear Perspex (Fig. 7A). The constant rectangular tunnel was designed with a cross-sectional dimension of 0.3 m×0.3 m, sufficient space for aerobatic maneuvers and the application of strong in-flight disturbances that are relevant to normal outdoor conditions. In the foraging area, an array of artificial linalool-scented nectar flowers was provided for the bees to feed. Authentic flower pollen was also provided for collection adjacent to the artificial flowers to ensure natural sustainment of the hive.

**Fig. 8.**
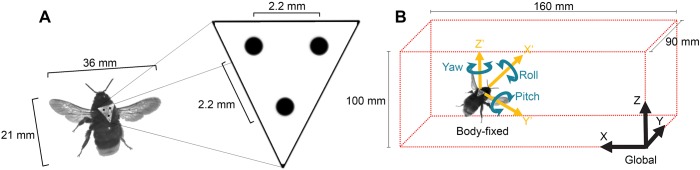
**Tracking details and coordinate systems.** (A) Layout of triangular markers affixed to the thorax of bees. Markers were aligned with longitudinal axis of bees such that the long arm always faced rearwards. Bumblebee dimensions are averages based on DLT measurements taken during steady flight. (B) Global and body-fixed coordinate systems defined within the calibrated volume.

Following a several-day habituation period, 50 healthy foraging bees were captured and cold-anesthetized. Markers (Fig. 8A) were then affixed to each of the bees according to the method described in Ravi et al. (2013). Flight was not impaired in any noticeable way by the markers or by the process of adding markers. Marked bees were later released back into the foraging area where they were able to fully recover and resume regular transit between the foraging area and the hive.

Flights through the Perspex tunnel were perturbed by a strong wind gust in the form of a thin, high-velocity air sheet inserted into the middle of the tunnel length. Gusts were directed along the cross-section positioned perpendicular to the longitudinal axis of the tunnel and were operated continuously during experimentation (see Movie 1 for visual representation of setup). Airflow resembling a gust (a net flow of air moving in a particular direction) was produced using a plastic air-knife nozzle driven by a Teral VFZ 1000 W ring blower with a restricted inlet. The nozzle outlet was designed such that it could be inserted flush with the inner surface of any of the four walls comprising the tunnel (Fig. 7B), thereby allowing us to easily change the gust airflow direction in any of the 90° positions without affecting the structure of the gust. On the face of the tunnel opposite to the gust, a meshed gap was created to allow air to escape and prevent flow recirculation. The strength of the gust was adjusted to contain an average flow velocity that applied a high-impulse force to flying bees without causing surface contact or loss of control leading to a crash.

Statistical significance in the results was determined by applying paired *t*-tests between sets of data. In all statistical tests, the sample size of each group was 10 bees (*n*=10). Probability values below a significance threshold of 0.05 were used to reject null hypotheses.

### Flow field characterization

The gust was quantified using particle image velocimetry (PIV) calculated from imagery produced by a Photron (Tokyo, Japan) high speed camera. With the gust in position, a pulsed laser sheet was projected along the middle of the tunnel such that a horizontal cross-section of the tunnel was illuminated. PIV measurements were conducted in multiple planar locations on the vertical axis spanning the total height of the gust (see Movie 1). Particles from evaporated olive oil were used to seed the air comprising the flow of the gust.

**Fig. 9.**
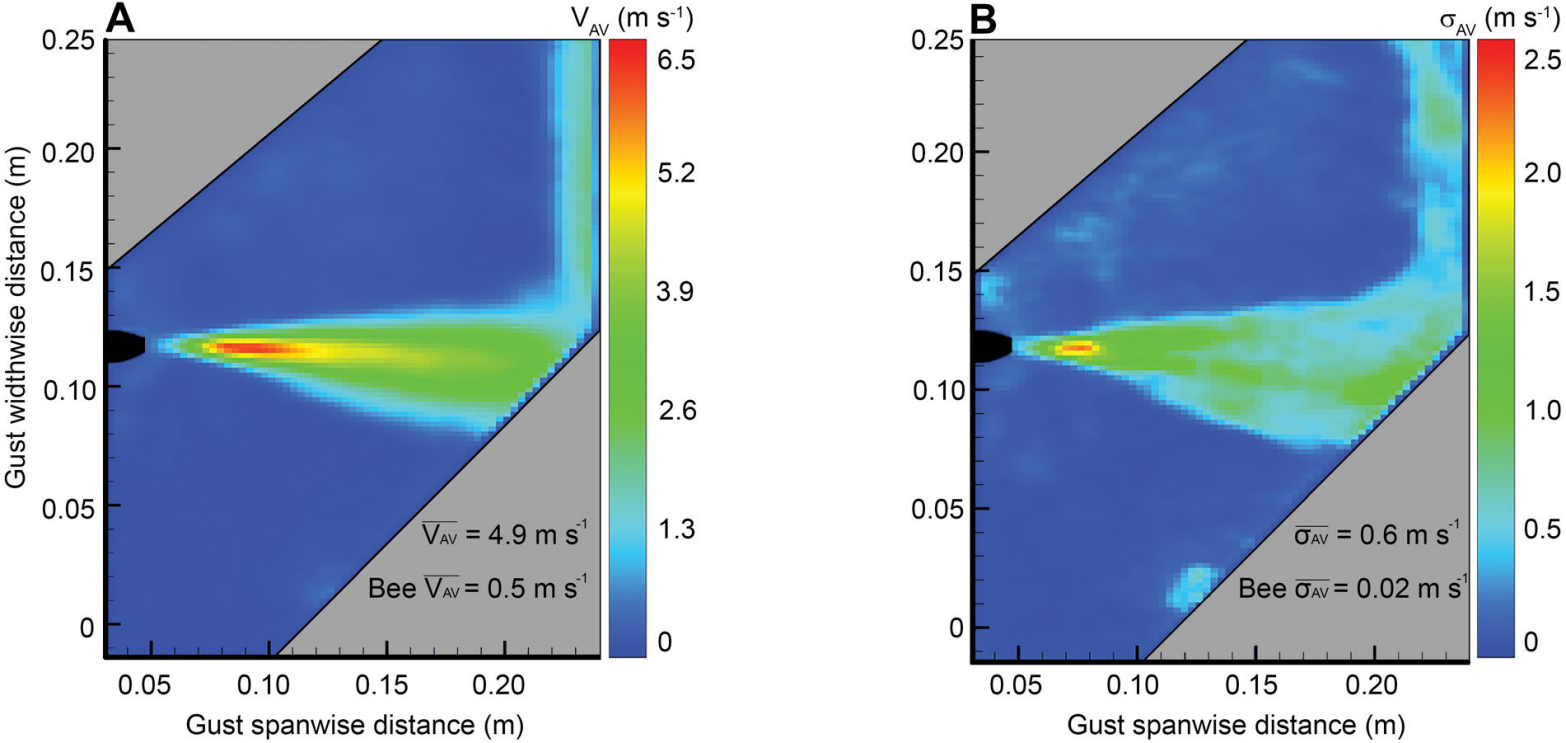
**Gust details.** Digital quantification of the gust showing (A) average gust velocity calculated from 300 samples captured in one second and (B) standard deviation of the gust velocity. The 2D images shown here resemble a plane orthogonal to the gust plane depicted in Fig. 1. To capture the gust in its actual position of operation (in the flight tunnel), a laser sheet was projected diagonally into the cross-sectional field of interest in the gust midpoint. Hence, resultant images are cropped in a slanted format to capture the structure of the gust. Grey shaded zones are the regions which were not illuminated by the diagonally slanted laser sheet and contain no useful data. The color bar label for the left image, V_AV_, is defined as the average velocity magnitude for each pixel sampled over 300 images corresponding to a gust duration of 1 s. The color bar label for the right image, σ_AV_, is defined as the standard deviation of average velocity magnitudes for the same sample of 300 images (Table 1). In Panel A, 
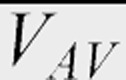
 represents the average gust velocity within the regions of the jet depicted in the image. Bee 
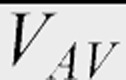
 is shown below this for comparison and represents the average forward velocity of bee in steady flight just before meeting the gust (gust entry velocity). In Panel B, 
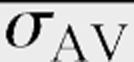
 represents the mean of all standard deviations of average gust velocity within the gust flow depicted in the contour image. Bee 
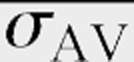
 is shown to provide an idea of how bee mean velocity changes during steady flight.

Gusts penetrated the stagnant air in the flight tunnel/interrogation volume at a mean velocity of approximately 4.9 m s^−1^ (Fig. 9A). The mean velocity profile extending vertically up the tunnel cross-section varied minimally, indicating that the gust was reasonably consistent. Maximum jet intensity near the outlet (approximately 20 mm from the tip of the outlet) was approximately 2.8 m s^−1^ greater than the mean flow elsewhere in the gust. The averaged boundaries at the edges of the gust flow had a constant linear form from the outlet point to the exit aperture on the opposite wall. The gust sheet tapered out by 8°, causing a linear change in the horizontal width of the gust. On average, the gust width grew from 3 mm to 45 mm across the width of the tunnel. Shear layer fluctuations occurred at a high frequency (at least 60 Hz) and the width of these unsteady movements varied by up to 6 mm (at the center axis of the tunnel). Relative to the rate at which bees travelled through the tunnel, the shear layer fluctuations buffeted bees rapidly (at least 10 times during their flight) and thus we consider the effects of spatial and temporal implications caused by the shear layer on the dynamic response negligible. To know precisely when bees penetrated the gust sheet, gust boundaries were defined by the average edge of the shear layer fluctuations across the entire width of the tunnel. The observed average forward flight velocity of bees was 0.50 m s^−1^ and the average body length was 21 mm. Error in the exact location of gust penetration caused by shear layer fluctuations was in the order of 2–3 mm (1/10th of the average bee body length). Gusts positioned in three different 90° positions were all identical in form, and the flow exiting at the opposite aperture in the wall did not recirculate or affect the structure of the gusts in the calibrated region of any of the three directions.

**Table 1.**
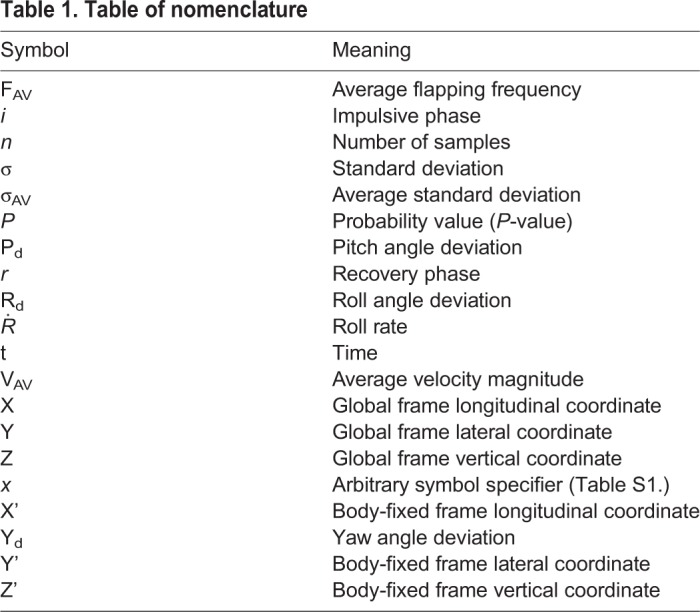
**Table of nomenclature**

### Analysis of flight motions

Bees were filmed using three Photron high-speed cameras recording 2000 fps at a shutter speed of 1/5000 s. Recordings were captured manually using a remote trigger as marked bees flew through the gust. The interrogation volume was defined by a rectangular prism of dimensions (160 mm×90 mm×100 mm) (Fig. 8B) which served as a calibration frame for direct linear transformation (DLT). The entire free-flight dynamic response of bees to gusts was encapsulated within the interrogation volume.

Spatial data of bees were extracted from the recordings using DLTdv5; an open-source MATLAB-executed software which tracks positional information of markers via DLT (Hedrick, 2008). Each of the three points on the markers was tracked in the global reference frame after down-sampling the data to a frequency of 1000 fps. Data were subsequently translated into a body reference frame by taking the rearmost point of the affixed marker on the bee as the origin. These data were smoothed using a fourth order low-pass Butterworth filter with a cut-off frequency of 30 Hz. Translational and rotational rates were calculated by performing numerical differentiation on the filtered positional data in the global and body-fixed reference frames. First derivative rates were smoothed using a fourth order Butterworth filter with a cut-off frequency of 15 Hz. Translational and rotational acceleration was then calculated by numerically differentiating the smoothed first derivative data (for more details see Fig. S1 and Fig. S2).

To determine the time at which bees began to enter the region of air impinged by the gust, we referred to our PIV interpretation of the structure and form of the gust. Drawing from this, it was estimated that the shear-layer fluctuated negligibly and that linear gust boundaries were clearly defined. This allowed us to produce a static 3D reconstruction of the gust in the same coordinate system used to measure and track bees. Thus, at each instance throughout the recorded flight of each bee, we were able to use this model to determine the gust position relative to the bee.

Error between our deduced prediction of the gust location and the absolute size and magnitude of its actual shear layer fluctuations was gauged by comparing our spatial calculations of the gust to the dynamic information extracted from bees. Accounting for body length and determining the locations at which accelerations in the direction of the gust spiked, we were able to see where bees began to be perturbed by the gust. We compared both of these methods of determining gust location and confirmed that discrepancies were within an order of millimeters (µ=2.75 mm).

Other sources of error were inherently drawn from the method of manually tracking dynamics from the calibrated and synchronized cameras. We calibrated the three cameras at the beginning and end of each set of flight recordings to minimize the likelihood of inaccurate DLT transformations caused by accidental bumps to the camera positions. To measure and control this digitization error we limited the DLT error residual to a maximum value of 1 pixel (1 pixel was usually about 0.2 mm corresponding to about 1/100 bee lengths). This also accounted for digitization error in the process of marker tracking which was limited by the number of pixels present in the recorded images. Marker views having a difficult or distant perspective in frames where the bee was at a difficult angle or made up of a smaller number of pixels (due to a low-flying bee), were difficult to pinpoint exactly on the actual marker centroid. For these frames, tracking was achieved manually on a frame-by-frame basis. This limited the contribution of inaccuracy due to residual error, allowing us to control the degree of error in the setup.

Impulse and recovery flight phases were distinguished by tracking the individual maneuvers carried out by bees. Following the sequence of impulsive maneuvers (i.e. those moving the body in the direction of the gust), the recovery phase was said to begin. To determine the time at which this occurred, the respective attitude acceleration curve was used to find the instant at which bees began to undergo motion in the corresponding recovery direction. This procedure of detecting the start of recovery produces phase durations that do not differ significantly when the calculated start time differs by a generous error estimate of ±10 frames.

## Supplementary Material

Supplementary information

First Person interview
