# Supplementary material for: Bees with attitude: the effects of directed gusts on flight trajectories
Source: Biol Open. 2018 Aug 22;7(10):bio034074. doi: 10.1242/bio.034074 (PMC6215418; doi:10.1242/bio.034074)
Supplement: Supplementary information [file biolopen-7-034074-s1.pdf]

Supplementary information

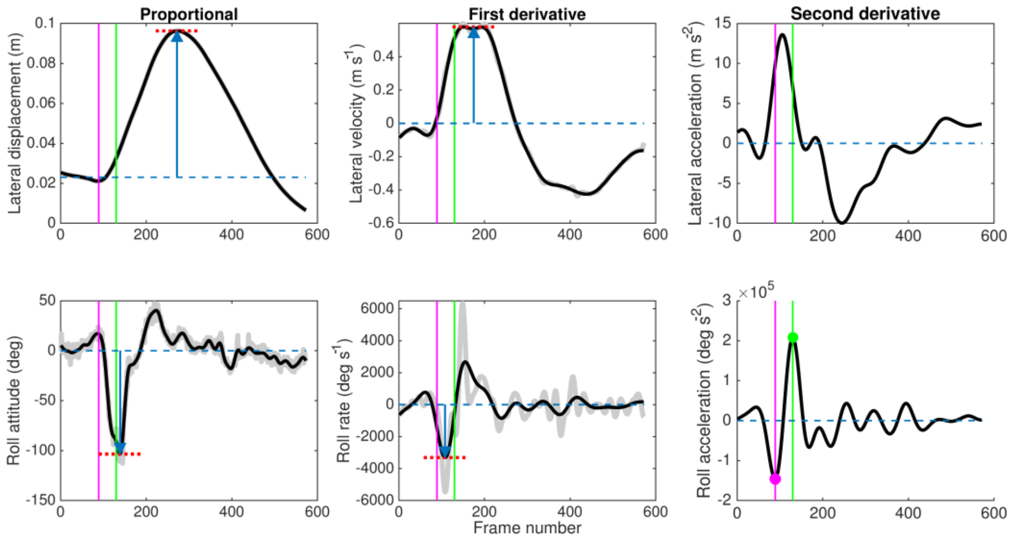

Fig. S1. Tracked marker dynamic information plotted against time (frame number) for an example bee flight. (A) Unfiltered data (light line) with the effect of the smoothing process shown by overlayed filtered data (dark line) for translational and rotational proportional data. Vertical lines represent the beginning of the impulsive phase (purple) and the recovery phase (green). Dotted red lines show how maximum dynamics values were calculated in this study. (B) Numerically differentiated data overlayed on raw unfiltered data. An additional filter (cutoff frequency 15 hz) was applied to first derivative data to smoothen the result (dark line). (C) Double-derivative data plotted against time. Circles show how commencement of the impulsive phase (purple circle) and the recovery phase (green) was identified where the centroids of the circles occur at the respective peaks of the second derivative of the roll acceleration curve.

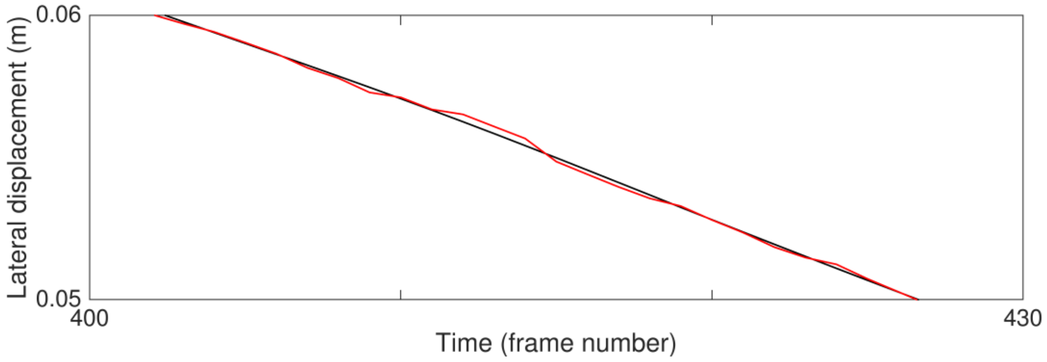

Fig. S2. Close-up window of tracked marker plotted against time (frame number) for an example bee flight. Filtered data is shown in black and the raw unfiltered data is plotted in red. All subsequent derivative data and attitude calculations were taken from the smoothed curve shown here.

Table S1. Means and standard errors of maximum dynamic changes used for the hypothesis tests on dynamic information for the four flow conditions used in this study.

Dynamics statistics								
	Sideward		Upward		Downward		No gust	
	Mean	Error (±)	Mean	Error (±)	Mean	Error (±)	Mean	Error (±)
Maximum roll angle deviation (deg)	54.34	4.93	42.3	5.59	40.1	12.29	18.70	3.63
Maximum pitch angle deviation (deg)	34.1	4.40	32.8	2.25	36.5	3.82	31.43	2.57
Maximum yaw angle deviation (deg)	37.7	6.42	23.3	7.72	29.92	7.05	6.82	4.38
Maximum Y deviation (m)	0.048	0.003	0.031	0.003	0.028	0.002	0.023	0.004
Maximum Z deviation (m)	0.054	0.008	0.032	0.002	0.053	0.006	0.009	0.002
Impulse time (t_i) (s)	0.072	0.01	0.064	0.01	0.059	0.01	-	-
Recovery time (t_r) (s)	0.121	0.04	0.051	0.05	0.125	0.03	-	-
Maximum roll rate ($\dot{R}$) (deg s^{-1})	2311	254	1514	133	1684	209	1599	596
Maximum pitch rate ($\dot{P}$) (deg s^{-1})	1007	234	1045	89	1058	93	458	129
Maximum yaw rate ($\dot{Y}$) (deg s^{-1})	1092	82	783	230	696	532	672	182
Minimum X velocity ($\dot{X}$) (m s^{-1})	0.13	0.04	0.17	0.03	0.07	0.02	0.401	0.047
Maximum Y velocity ($\dot{Y}$) (m s^{-1})	0.43	0.03	0.21	0.03	0.13	0.04	0.142	0.040
Maximum Z velocity ($\dot{Z}$) (m s^{-1})	0.19	0.026	0.20	0.024	0.44	0.039	0.077	0.011

Table S2. p-values from hypothesis tests on each comparison of dynamic information for the four flow conditions used in this study.

p-values of comparisons of dynamics statistics						
Proportional dynamics	$x_{\mathrm{side}}-x_{\mathrm{up}}$	$x_{\mathrm{side}}-\mathrm{p}_{\mathrm{ng}}$	$x_{\mathrm{up}}-x_{\mathrm{down}}$	$x_{\mathrm{up}}-x_{\mathrm{ng}}$	$x_{\mathrm{down}}-x_{\mathrm{side}}$	$x_{\mathrm{down}}-x_{\mathrm{ng}}$
Maximum roll deviation	0.004	< 0.001	0.224	< 0.001	0.236	0.001
Maximum pitch deviation	0.304	0.021	0.004	0.002	0.261	< 0.001
Maximum yaw deviation	0.012	< 0.001	0.260	0.006	0.001	< 0.001
Maximum Y deviation	0.008	0.002	0.580	0.794	0.077	0.454
Maximum Z deviation	0.343	0.017	0.002	0.008	< 0.001	0.006
Impulse time (t_i)	0.915	-	0.672	-	0.525	-
Recovery time (t_r)	0.012	-	0.001	-	0.816	-
Maximum roll rate ($\dot{R}$)	0.059	0.001	0.376	0.019	0.977	0.089
Maximum pitch rate ($\dot{P}$)	0.413	0.038	0.943	0.011	0.558	0.045
Maximum yaw rate ($\dot{Y}$)	0.268	0.040	0.561	0.052	0.561	0.180
Minimum X velocity ($\dot{X}$)	0.429	< 0.001	0.037	0.000	0.246	< 0.001
Maximum Y velocity ($\dot{Y}$)	< 0.001	< 0.001	< 0.001	0.014	0.785	< 0.001
Maximum Z velocity ($\dot{Z}$)	0.197	< 0.001	< 0.001	< 0.001	< 0.001	< 0.001

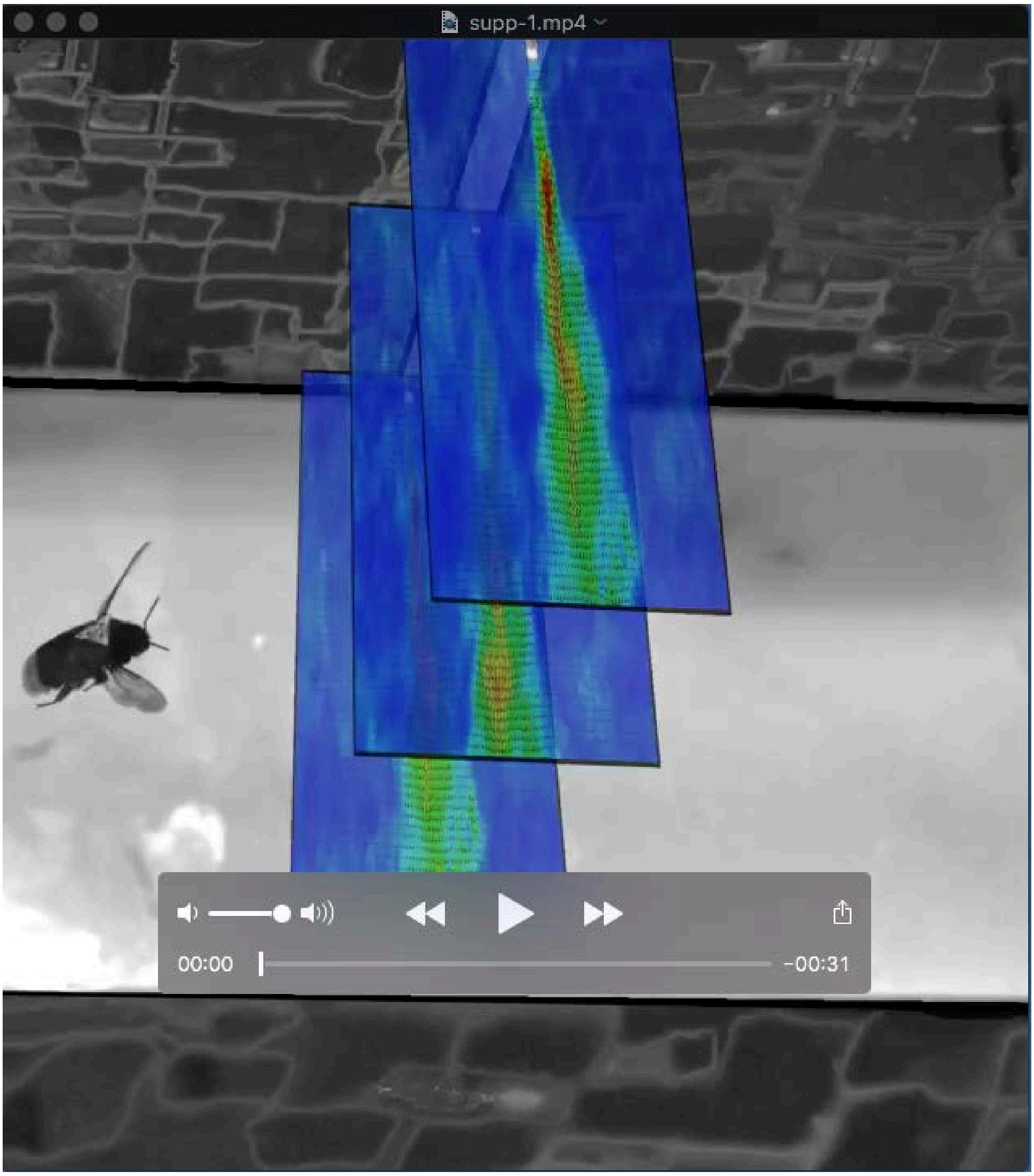

Movie S1. Example recording of a bumblebee flying through a sideward gust. 2D reconstructed PIV measurements of the gust taken in three different planes are displayed in the perspective of the camera as the bee travels through the flight tunnel. Images sampled across a one second gust duration (similar to the flight recording durations) provide a visual representation of how the gust varies as bees fly through it.
