# Supplementary material for: Bees with attitude: the effects of directed gusts on flight trajectories
Source: Biol Open. 2018 Aug 22;7(10):bio034074. doi: 10.1242/bio.034074 (PMC6215418; doi:10.1242/bio.034074)
Supplement: First Person interview [file biolopen-7-034074-s2.pdf]

FIRST PERSON

First person – Timothy Jakobi

First Person is a series of interviews with the first authors of a selection of papers published in Biology Open, helping early-career researchers promote themselves alongside their papers. Timothy Jakobi is first author on 'Bees with attitude: the effects of directed gusts on flight trajectories', published in BiO. Timothy is a PhD student in the lab of Simon Watkins at RMIT University, Victoria, Australia, investigating control strategies for small-scale flapping flight in complex environments.

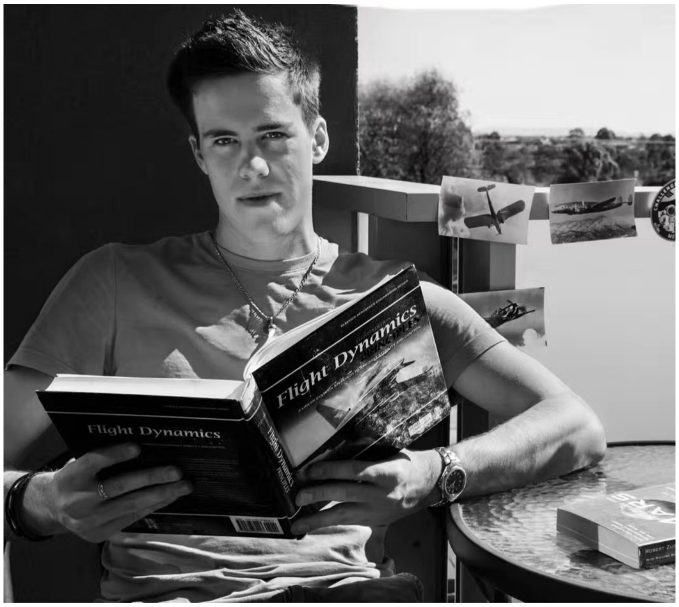

Timothy Jakobi

What is your scientific background and the general focus of your lab?

Over the duration of my undergraduate studies in aerospace engineering and also my industry experience at Rolls-Royce, I developed a strong interest in the flight of small unmanned aerial vehicles. At RMIT, our lab is centrally focussed on micro unmanned aerial vehicles, particularly the effects of atmospheric conditions on the flight of such systems. As I joined the Watkins lab to undertake PhD studies, my research interests focused on the ultra-small scale flight – that of the size of insects. At this scale, flapping wings seem to offer potential solutions to many of the challenges involved just as already demonstrated broadly by insects. I aim to uncover the mysterious aerodynamic interactions that occur at this scale when atmospheric phenomena strike flapping wings along with the associated potential control strategies that may aid flight in this domain.

How would you explain the main findings of your paper to non-scientific family and friends?

As we look outside, it is not difficult to spot the brilliant flying capabilities of insects and small birds. During times of rain and even gale-force winds, we see that many insects still manage to carry on with their habitual activities; although this may be overlooked because we are so very used to sighting these marvels of nature. The knowledge that insects are able to conquer these adverse winds means that there is good reason to uncover the aerodynamics and flight mechanics which facilitate it. This paper presents results that quantify the effects of gusts on the flight kinematics of bees, allowing us to understand more about potential control strategies for flight at this scale which could be of benefit to the future development of smaller drones.

What are the potential implications of these results for your field of research?

The results presented in this paper show that gusts coming from particular directions can induce significantly worse flight perturbations than other directions to flying insects. This suggests that the aerodynamics present upon the wing are affected uniquely by gusts and that they are dependent on the direction of the gust. This research pursues an important consideration for the flight of future flying robots, whereby flight in certain atmospheric flows can have negative consequences on the performance of a flying platform. These air conditions may present serious implications for control, and the best methods of overcoming them are likely those which are displayed by the magnificent insects analysed and discussed in these works.

> **"Through embarking on this research I definitely hold a greater respect and awe for nature's widespread biological marvels that I never previously anticipated."**

What has surprised you the most while conducting your research?

The most fascinating aspect of conducting this research was the learning process while working with live organisms. The sheer magnificence of bumblebee flight and their behavioural patterns surprised me the most. The experimental nature of this work made me examine bumblebees in a new perspective and examine their remarkable capabilities in pristine detail. This led me to a realisation that bumblebees and probably other volant creatures are far more intelligent than I ever previously realised. The relentless efforts bees undergo to sustain their routine and continually support their fellow colony through our complex maze of disturbance-ridden tunnels was astonishing. For example, after learning the location of food and pollen, bees quickly established heavy traffic to and from the source, displaying both fast responses to our inflight perturbations and workaround behaviours when something in their environment was changed. Through embarking on this research I definitely hold a greater respect and awe for nature's widespread biological marvels that I never previously anticipated.

Timothy Jakobi's contact details: School of Aerospace Mechanical and Manufacturing Engineering, RMIT University, GPO Box 2476, Melbourne, Victoria 3001, Australia.
E-mail: tim@timjakobi.org

Biology Open (2018): doi:10.1242/bio.034074: Supplementary information

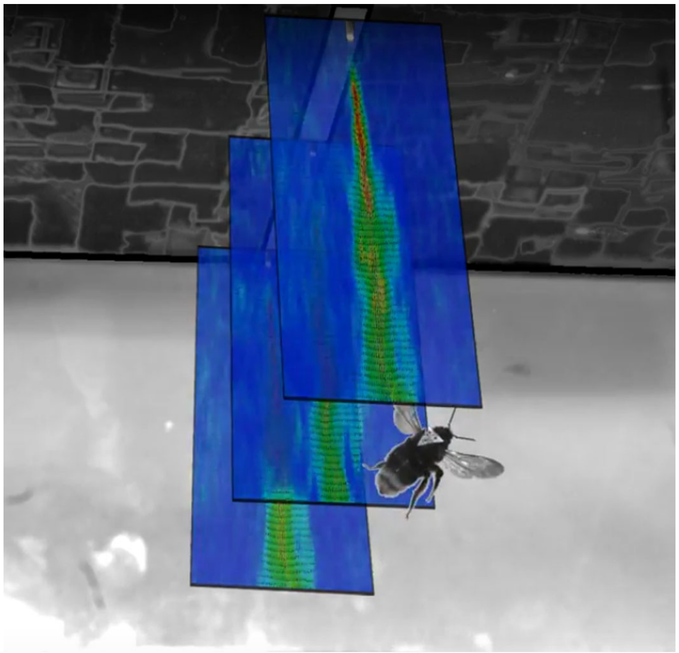

Bumblebee encountering a gust as it flies through a complex aerial environment.

What, in your opinion, are some of the greatest achievements in your field and how has this influenced your research?

The leading edge vortex is perhaps the most crucial element of flapping flight at small scales. Its discovery (not much longer than two decades ago) provides explanations where conventional aerodynamics don't hold up and it marked an important milestone beyond which subsequent studies have really built the core knowledge of flapping flight that currently stands today. Other aerodynamic mechanisms that operate on flapping insect wings are linked in one way or another with the leading edge vortex and are equally remarkable. Put together, the many complexities inherent to flight biomechanics and coupled fluid mechanical interactions are extraordinary. My research is influenced by a constant motivation to feed information that helps to piece together the complexities of such an elaborate system of systems, specifically about how flapping wings can operate within complex environments the way they are observed in biological examples.

> "I think it's important to hold a true passion for the work that we try to fulfil, regardless of its perceived importance or its value made by the opinion of others."

What changes do you think could improve the professional lives of early-career scientists?

I think it's important to hold a true passion for the work that we try to fulfil, regardless of its perceived importance or its value made by the opinion of others. Sometimes the love for what we do is lost due to competing stresses or other things that can sap energy from a task at hand. If something happens, I think it's important to step back and take a break because this can help renew the energy that needs to be applied to figure something out. Time spent working on an alternative task is a good way to both finish smaller projects and renew dormant motivation on a larger goal. I also find that sharing work with other people face-to-face is extremely useful in helping clarify the most difficult parts of a problem. On many occasions I've been mentally blocked on a problem for days on end and in the end all it took was a simple conversation with a colleague to land on a solution.

What's next for you?

I will finish my PhD within the next year. Afterwards, I hope to get into a position where I can continue this line of research and potentially take it further. I'm particularly interested in applying bio-inspired design to aerospace systems, so I'd like to have an influence on the development of novel flight platforms and drone control mechanisms in the future, especially those approaching the scale insects.

Reference

Jakobi, T., Kolomenskiy, D., Ikeda, T., Watkins, S., Fisher, A., Liu, H. and Ravi, S. (2018). Bees with attitude: the effects of directed gusts on flight trajectories. *Biol. Open* **7**: bio034074.
